# 1,1′,2,2′,3,3′,4,4′-Octa­methyl­ferro­cenium 2,5-dibromo-4-hy­droxy-3,6-dioxocyclo­hexa-1,4-dien-1-olate

**DOI:** 10.1107/S1600536811025499

**Published:** 2011-07-02

**Authors:** Tomoyuki Mochida

**Affiliations:** a Department of Chemistry, Graduate School of Science, Kobe University, Rokkodai, Nada, Hyogo 657-8501, Japan

## Abstract

In the title salt, octa­methyl­ferrocenium bromanilate, [Fe(C_9_H_13_)_2_](C_6_HBr_2_O_4_), the Fe atom and the bromanilate anion lie on a mirror plane. The octa­methyl­ferrocenium cation adopts an eclipsed conformation. An intra­molecular O—H⋯O hydrogen bond is present in the bromanilate anion. In the crystal, the cations and anions are stacked alternately, forming a one-dimensional columnar structure along [010].

## Related literature

For general background to ferrocene-based charge-transfer complexes, see: Miller *et al.* (1994 ▶); Mochida *et al.* (2007 ▶). For organometallic supra­molecular compounds, see: Braga *et al.* (2001 ▶); Horikoshi *et al.* (2004 ▶). For phase transitions in octa- and deca­methyl­ferrocene complexes, see: Mochida *et al.* (2011 ▶); Mochida & Yoza (2010 ▶). For related structures containing bromanilic acid, see: Mochida (2010 ▶); Thomas *et al.* (2009 ▶); Tomura & Yamashita (2000 ▶); Zaman *et al.* (2001*a* ▶,*b* ▶, 2004 ▶). For the structure of the octa­methyl­ferrocenium cation, see: Miller *et al.* (1989 ▶).
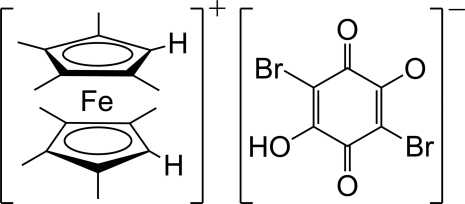


## Experimental

### 

#### Crystal data

[Fe(C_9_H_13_)_2_](C_6_HBr_2_O_4_)
*M* *_r_* = 595.13
Orthorhombic, 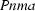

*a* = 14.7106 (15) Å
*b* = 10.4938 (11) Å
*c* = 14.9461 (15) Å
*V* = 2307.2 (4) Å^3^
*Z* = 4
Mo *K*α radiation
μ = 4.15 mm^−1^
*T* = 173 K
0.38 × 0.08 × 0.08 mm

#### Data collection

Bruker APEX CCD diffractometer
Absorption correction: multi-scan (*SADABS*; Sheldrick, 1996 ▶) *T*_min_ = 0.306, *T*_max_ = 0.746
14287 measured reflections
2804 independent reflections
1699 reflections with *I* > 2σ(*I*)
*R*_int_ = 0.087

#### Refinement

*R*[*F* ^2^ > 2σ(*F* ^2^)] = 0.041
*wR*(*F* ^2^) = 0.084
*S* = 1.00
2804 reflections
167 parameters
H atoms treated by a mixture of independent and constrained refinement
Δρ_max_ = 0.56 e Å^−3^
Δρ_min_ = −0.39 e Å^−3^

### 

Data collection: *SMART* (Bruker, 2007 ▶); cell refinement: *SAINT* (Bruker, 2007 ▶); data reduction: *SAINT*; program(s) used to solve structure: *SHELXS97* (Sheldrick, 2008 ▶); program(s) used to refine structure: *SHELXL97* (Sheldrick, 2008 ▶); molecular graphics: *SHELXTL* (Sheldrick, 2008 ▶); software used to prepare material for publication: *SHELXTL*.

## Supplementary Material

Crystal structure: contains datablock(s) I, global. DOI: 10.1107/S1600536811025499/hy2444sup1.cif

Structure factors: contains datablock(s) I. DOI: 10.1107/S1600536811025499/hy2444Isup2.hkl

Additional supplementary materials:  crystallographic information; 3D view; checkCIF report

## Figures and Tables

**Table 1:** Hydrogen-bond geometry (Å, °)

*D*—H⋯*A*	*D*—H	H⋯*A*	*D*⋯*A*	*D*—H⋯*A*
O2—H1⋯O1	1.01 (8)	1.70 (9)	2.534 (5)	137 (7)

**Fig. 1.**
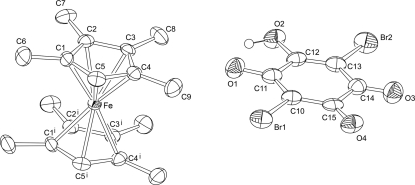
The molecular structure of the title compound with displacement ellipsoids drawn at the 50% probability level. H atoms except for the hydroxyl group are omitted for clarity. [Symmetry code: (i) x, 1/2-y, z.]

**Fig. 2.**
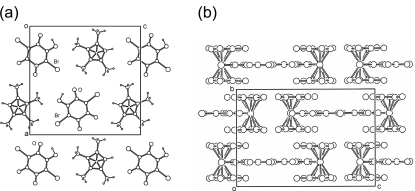
Packing diagrams of the title compound, (a) viewed along the b-axis and (b) viewed along the a-axis. H atoms are omitted for clarity in (b).

## supplementary crystallographic information

### Comment

Organometallic charge-transfer salts (Miller *et al.*, 1994; Mochida *et al.*, 2007) and organometallic hydrogen-bonded supramolecular compounds (Braga *et al.*, 2001, Horikoshi *et al.*, 2004) have attracted attention mainly from the viewpoints of physical properties and crystal engineering, respectively. This paper reports the crystal structure of the title compound, a charge-transfer salt of octamethylferrocene with bromanilic acid. Bromanilic acid acts as proton donor and electron acceptor, and is useful for building organometallic complexes (Mochida, 2010; Thomas *et al.*, 2009; Tomura & Yamashita, 2000; Zaman *et al.*, 2001*a*, 2004).

The title compound consists of octamethylferrocenium cation and bromanilate anion, which is a deprotonated form of bromanilic acid (Fig. 1). The asymmetric unit contains half of the cation and half of the anion, as the Fe atom and bromanilate anion lie on a mirror plane. The Fe—C(Cp) distances in the cation are 2.072 (3)–2.120 (3) Å (average: 2.102 Å), which is a typical value of the octamethylferrocenium cation (Miller *et al.*, 1989). The C_5_HMe_4_ groups of the cation adopt an eclipsed conformation and exhibit no disorder. In the anion, the C—O bond length to the deprotonated O atom is shortened compared to that to the protonated O atom [1.234 (6) *versus* 1.337 (6) Å]. In the crystal, the cations and anions are alternately stacked along the *b*-axis to form one-dimensional columns (Fig. 2). There are no intermolecular hydrogen bonds, whereas the bromanilate anion shows an intramolecular O—H···O hydrogen bond (Table 1). The local molecular arrangement closely resembles that of a decamethylferrocene–bromanilate compound (Zaman *et al.*, 2011*b*), although they are not isomorphous. In both crystals, the electronegative atoms (O or Br) of the anion appear to surround the Fe atoms of the cation, leading to the co-planar arrangement of the Fe atoms and the anion planes, as seen in Fig. 2b. In this compound, no O···Br close contacts are seen, in contrast to decamethylferrocene–bromanilate compound, and C—H···Br contacts are observed between the cation and anion (H···Br distance = 3.01 Å).

DSC measurements revealed no traces of phase transitions between 120–400 K, whereas decamethyl- and octamethylferrocene complexes often undergo phase transitions associated with order-disorder of the cyclopentadienyl rings (Mochida *et al.*, 2011; Mochida & Yoza, 2010). An exothermic peak corresponding to decomposition was observed around 408 K.

### Experimental

Violet needle crystals of the title compound were grown by slow evaporation of solvent from a 1:1 solution of octamethylferrocene and bromanilic acid in dichloromethane at 223 K. IR (KBr, cm^-1^): 2919, 1642, 1562, 1368, 1336, 1167, 1030, 957, 790, 553.

### Refinement

The hydroxyl H atom was identified in a difference Fourier map and allowed to refine isotropically. Other H atoms were placed at calculated positions and refined in a riding model, with C—H = 0.98 (methyl) and 1.00 (aromatic) Å and with *U*_iso_(H) = 1.2(1.5 for methyl)*U*_eq_(C).

### Crystal data

**Table:**

[Fe(C_9_H_13_)_2_](C_6_HBr_2_O_4_)	*F*(000) = 1196
*M**_r_* = 595.13	*D*_x_ = 1.713 Mg m^−^^3^
Orthorhombic, *P**n**m**a*	Mo *K*α radiation, λ = 0.71073 Å
Hall symbol: -P 2ac 2n	Cell parameters from 1436 reflections
*a* = 14.7106 (15) Å	θ = 2.7–24.0°
*b* = 10.4938 (11) Å	µ = 4.15 mm^−^^1^
*c* = 14.9461 (15) Å	*T* = 173 K
*V* = 2307.2 (4) Å^3^	Needle, violet
*Z* = 4	0.38 × 0.08 × 0.08 mm

### Data collection

**Table:**

Bruker APEX CCD diffractometer	2804 independent reflections
Radiation source: fine-focus sealed tube	1699 reflections with *I* > 2σ(*I*)
graphite	*R*_int_ = 0.087
φ and ω scans	θ_max_ = 27.5°, θ_min_ = 1.9°
Absorption correction: multi-scan (*SADABS*; Sheldrick, 1996)	*h* = −19→18
*T*_min_ = 0.306, *T*_max_ = 0.746	*k* = −13→13
14287 measured reflections	*l* = −19→15

### Refinement

**Table:**

Refinement on *F*^2^	Primary atom site location: structure-invariant direct methods
Least-squares matrix: full	Secondary atom site location: difference Fourier map
*R*[*F*^2^ > 2σ(*F*^2^)] = 0.041	Hydrogen site location: inferred from neighbouring sites
*wR*(*F*^2^) = 0.084	H atoms treated by a mixture of independent and constrained refinement
*S* = 1.00	*w* = 1/[σ^2^(*F*_o_^2^) + (0.032*P*)^2^] where *P* = (*F*_o_^2^ + 2*F*_c_^2^)/3
2804 reflections	(Δ/σ)_max_ = 0.001
167 parameters	Δρ_max_ = 0.56 e Å^−^^3^
0 restraints	Δρ_min_ = −0.39 e Å^−^^3^

### Fractional atomic coordinates and isotropic or equivalent isotropic displacement parameters (Å^2^)

**Table:**

	*x*	*y*	*z*	*U*_iso_*/*U*_eq_	
Br1	0.62817 (4)	0.2500	0.60939 (4)	0.04543 (19)	
Br2	0.89861 (5)	0.2500	0.25105 (4)	0.0542 (2)	
Fe1	0.24413 (4)	0.2500	0.60312 (4)	0.02121 (17)	
C2	0.2127 (2)	0.0859 (3)	0.5266 (2)	0.0264 (8)	
C1	0.1616 (2)	0.0870 (3)	0.6081 (2)	0.0266 (8)	
C5	0.2252 (2)	0.0881 (3)	0.6802 (2)	0.0298 (8)	
H5	0.2094	0.0869	0.7453	0.036*	
C3	0.3072 (2)	0.0853 (3)	0.5494 (2)	0.0256 (8)	
C4	0.3147 (2)	0.0867 (3)	0.6443 (2)	0.0285 (8)	
C8	0.3846 (2)	0.0811 (4)	0.4848 (2)	0.0410 (10)	
H8A	0.4334	0.1372	0.5058	0.062*	
H8B	0.3637	0.1097	0.4259	0.062*	
H8C	0.4074	−0.0064	0.4803	0.062*	
C7	0.1734 (3)	0.0829 (3)	0.4341 (2)	0.0389 (10)	
H7A	0.1633	−0.0057	0.4160	0.058*	
H7B	0.2157	0.1236	0.3923	0.058*	
H7C	0.1154	0.1288	0.4335	0.058*	
C9	0.4003 (2)	0.0809 (4)	0.6978 (3)	0.0420 (10)	
H9A	0.4187	−0.0083	0.7052	0.063*	
H9B	0.3899	0.1192	0.7567	0.063*	
H9C	0.4484	0.1278	0.6666	0.063*	
C6	0.0605 (2)	0.0820 (3)	0.6176 (2)	0.0415 (10)	
H6A	0.0323	0.1336	0.5705	0.062*	
H6B	0.0430	0.1155	0.6763	0.062*	
H6C	0.0399	−0.0065	0.6121	0.062*	
C12	0.7307 (4)	0.2500	0.3449 (3)	0.0328 (13)	
C10	0.7062 (4)	0.2500	0.5083 (3)	0.0324 (13)	
C11	0.6678 (4)	0.2500	0.4242 (3)	0.0357 (13)	
C15	0.8004 (4)	0.2500	0.5240 (4)	0.0332 (13)	
C14	0.8635 (4)	0.2500	0.4391 (4)	0.0365 (13)	
C13	0.8211 (4)	0.2500	0.3525 (3)	0.0366 (13)	
O1	0.5845 (3)	0.2500	0.4037 (2)	0.0482 (10)	
O4	0.8385 (3)	0.2500	0.5975 (2)	0.0473 (11)	
O3	0.9454 (3)	0.2500	0.4508 (3)	0.0532 (11)	
O2	0.6862 (3)	0.2500	0.2669 (2)	0.0457 (11)	
H1	0.625 (5)	0.2500	0.297 (6)	0.13 (3)*	

### Atomic displacement parameters (Å^2^)

**Table:**

	*U*^11^	*U*^22^	*U*^33^	*U*^12^	*U*^13^	*U*^23^
Br1	0.0608 (5)	0.0432 (4)	0.0323 (3)	0.000	0.0033 (3)	0.000
Br2	0.0666 (5)	0.0589 (4)	0.0373 (4)	0.000	0.0083 (3)	0.000
Fe1	0.0223 (4)	0.0178 (3)	0.0235 (4)	0.000	−0.0008 (3)	0.000
C2	0.032 (2)	0.0177 (17)	0.030 (2)	−0.0013 (16)	−0.0022 (16)	−0.0020 (15)
C1	0.0208 (18)	0.0214 (18)	0.038 (2)	−0.0037 (14)	0.0036 (16)	0.0001 (17)
C5	0.040 (2)	0.0222 (19)	0.027 (2)	−0.0008 (16)	0.0022 (17)	0.0046 (15)
C3	0.027 (2)	0.0155 (17)	0.034 (2)	0.0029 (15)	0.0043 (15)	−0.0034 (16)
C4	0.031 (2)	0.0206 (19)	0.034 (2)	0.0040 (16)	−0.0047 (16)	−0.0004 (16)
C8	0.038 (3)	0.035 (2)	0.050 (3)	0.0073 (18)	0.0156 (19)	−0.003 (2)
C7	0.055 (3)	0.029 (2)	0.032 (2)	−0.0029 (19)	−0.0125 (18)	−0.0037 (18)
C9	0.040 (2)	0.035 (2)	0.051 (3)	−0.0002 (19)	−0.0165 (19)	0.005 (2)
C6	0.028 (2)	0.036 (2)	0.060 (3)	−0.0081 (18)	0.0086 (19)	0.003 (2)
C12	0.049 (4)	0.026 (3)	0.024 (3)	0.000	−0.010 (3)	0.000
C10	0.049 (4)	0.028 (3)	0.020 (3)	0.000	−0.001 (2)	0.000
C11	0.050 (4)	0.027 (3)	0.030 (3)	0.000	−0.006 (3)	0.000
C15	0.055 (4)	0.014 (3)	0.031 (3)	0.000	−0.006 (3)	0.000
C14	0.041 (4)	0.029 (3)	0.040 (3)	0.000	−0.004 (3)	0.000
C13	0.051 (4)	0.027 (3)	0.032 (3)	0.000	0.001 (3)	0.000
O1	0.046 (3)	0.055 (3)	0.043 (2)	0.000	−0.011 (2)	0.000
O4	0.064 (3)	0.045 (3)	0.033 (2)	0.000	−0.016 (2)	0.000
O3	0.048 (3)	0.060 (3)	0.051 (3)	0.000	−0.006 (2)	0.000
O2	0.060 (3)	0.053 (3)	0.024 (2)	0.000	−0.014 (2)	0.000

### Geometric parameters (Å, °)

**Table:**

Br1—C10	1.898 (5)	C8—H8B	0.9800
Br2—C13	1.897 (5)	C8—H8C	0.9800
Fe1—C5^i^	2.072 (3)	C7—H7A	0.9800
Fe1—C5	2.072 (3)	C7—H7B	0.9800
Fe1—C4^i^	2.096 (3)	C7—H7C	0.9800
Fe1—C4	2.096 (3)	C9—H9A	0.9800
Fe1—C1^i^	2.099 (3)	C9—H9B	0.9800
Fe1—C1	2.099 (3)	C9—H9C	0.9800
Fe1—C2^i^	2.119 (3)	C6—H6A	0.9800
Fe1—C2	2.119 (3)	C6—H6B	0.9800
Fe1—C3^i^	2.120 (3)	C6—H6C	0.9800
Fe1—C3	2.120 (3)	C12—C13	1.334 (7)
C2—C1	1.432 (4)	C12—O2	1.337 (6)
C2—C3	1.433 (5)	C12—C11	1.505 (7)
C2—C7	1.499 (4)	C10—C11	1.378 (7)
C1—C5	1.427 (5)	C10—C15	1.405 (7)
C1—C6	1.495 (4)	C11—O1	1.262 (6)
C5—C4	1.422 (5)	C15—O4	1.234 (6)
C5—H5	1.0000	C15—C14	1.571 (7)
C3—C4	1.421 (5)	C14—O3	1.218 (6)
C3—C8	1.493 (4)	C14—C13	1.437 (7)
C4—C9	1.493 (4)	O2—H1	1.01 (8)
C8—H8A	0.9800		
			
C5^i^—Fe1—C5	110.2 (2)	C1—C5—Fe1	71.03 (19)
C5^i^—Fe1—C4^i^	39.90 (13)	C4—C5—H5	125.6
C5—Fe1—C4^i^	125.03 (14)	C1—C5—H5	125.6
C5^i^—Fe1—C4	125.03 (14)	Fe1—C5—H5	125.6
C5—Fe1—C4	39.90 (13)	C4—C3—C2	108.2 (3)
C4^i^—Fe1—C4	109.68 (19)	C4—C3—C8	125.9 (3)
C5^i^—Fe1—C1^i^	40.01 (13)	C2—C3—C8	125.9 (3)
C5—Fe1—C1^i^	124.82 (13)	C4—C3—Fe1	69.39 (19)
C4^i^—Fe1—C1^i^	67.03 (14)	C2—C3—Fe1	70.21 (18)
C4—Fe1—C1^i^	160.47 (14)	C8—C3—Fe1	127.1 (2)
C5^i^—Fe1—C1	124.82 (13)	C3—C4—C5	107.8 (3)
C5—Fe1—C1	40.00 (13)	C3—C4—C9	126.8 (3)
C4^i^—Fe1—C1	160.47 (14)	C5—C4—C9	125.4 (3)
C4—Fe1—C1	67.03 (14)	C3—C4—Fe1	71.20 (19)
C1^i^—Fe1—C1	109.18 (18)	C5—C4—Fe1	69.14 (19)
C5^i^—Fe1—C2^i^	66.68 (13)	C9—C4—Fe1	127.5 (2)
C5—Fe1—C2^i^	159.65 (14)	C3—C8—H8A	109.5
C4^i^—Fe1—C2^i^	66.55 (13)	C3—C8—H8B	109.5
C4—Fe1—C2^i^	158.63 (14)	H8A—C8—H8B	109.5
C1^i^—Fe1—C2^i^	39.67 (12)	C3—C8—H8C	109.5
C1—Fe1—C2^i^	123.73 (13)	H8A—C8—H8C	109.5
C5^i^—Fe1—C2	159.65 (14)	H8B—C8—H8C	109.5
C5—Fe1—C2	66.68 (13)	C2—C7—H7A	109.5
C4^i^—Fe1—C2	158.63 (14)	C2—C7—H7B	109.5
C4—Fe1—C2	66.55 (13)	H7A—C7—H7B	109.5
C1^i^—Fe1—C2	123.73 (13)	C2—C7—H7C	109.5
C1—Fe1—C2	39.68 (12)	H7A—C7—H7C	109.5
C2^i^—Fe1—C2	108.75 (18)	H7B—C7—H7C	109.5
C5^i^—Fe1—C3^i^	66.45 (14)	C4—C9—H9A	109.5
C5—Fe1—C3^i^	159.74 (13)	C4—C9—H9B	109.5
C4^i^—Fe1—C3^i^	39.41 (13)	H9A—C9—H9B	109.5
C4—Fe1—C3^i^	124.10 (13)	C4—C9—H9C	109.5
C1^i^—Fe1—C3^i^	66.56 (13)	H9A—C9—H9C	109.5
C1—Fe1—C3^i^	158.65 (14)	H9B—C9—H9C	109.5
C2^i^—Fe1—C3^i^	39.52 (12)	C1—C6—H6A	109.5
C2—Fe1—C3^i^	123.65 (14)	C1—C6—H6B	109.5
C5^i^—Fe1—C3	159.74 (13)	H6A—C6—H6B	109.5
C5—Fe1—C3	66.45 (14)	C1—C6—H6C	109.5
C4^i^—Fe1—C3	124.10 (13)	H6A—C6—H6C	109.5
C4—Fe1—C3	39.41 (13)	H6B—C6—H6C	109.5
C1^i^—Fe1—C3	158.65 (14)	C13—C12—O2	124.2 (5)
C1—Fe1—C3	66.56 (13)	C13—C12—C11	123.1 (5)
C2^i^—Fe1—C3	123.66 (14)	O2—C12—C11	112.7 (5)
C2—Fe1—C3	39.52 (12)	C11—C10—C15	123.9 (5)
C3^i^—Fe1—C3	109.26 (18)	C11—C10—Br1	118.5 (4)
C1—C2—C3	107.8 (3)	C15—C10—Br1	117.6 (4)
C1—C2—C7	125.7 (3)	O1—C11—C10	128.3 (5)
C3—C2—C7	126.5 (3)	O1—C11—C12	114.0 (5)
C1—C2—Fe1	69.42 (18)	C10—C11—C12	117.8 (5)
C3—C2—Fe1	70.27 (18)	O4—C15—C10	126.6 (5)
C7—C2—Fe1	126.8 (2)	O4—C15—C14	116.8 (5)
C5—C1—C2	107.4 (3)	C10—C15—C14	116.6 (4)
C5—C1—C6	125.5 (3)	O3—C14—C13	123.9 (5)
C2—C1—C6	127.0 (3)	O3—C14—C15	118.0 (5)
C5—C1—Fe1	68.97 (18)	C13—C14—C15	118.1 (5)
C2—C1—Fe1	70.90 (18)	C12—C13—C14	120.6 (5)
C6—C1—Fe1	127.4 (2)	C12—C13—Br2	122.1 (4)
C4—C5—C1	108.8 (3)	C14—C13—Br2	117.4 (4)
C4—C5—Fe1	70.95 (19)	C12—O2—H1	92 (5)

Symmetry codes: (i) *x*, −*y*+1/2, *z*.

### Hydrogen-bond geometry (Å, °)

**Table:**

*D*—H···*A*	*D*—H	H···*A*	*D*···*A*	*D*—H···*A*
O2—H1···O1	1.01 (8)	1.70 (9)	2.534 (5)	137 (7)
